# Isolation and purification of polysaccharides from *Bupleurum marginatum Wall.ex DC* and their anti-liver fibrosis activities

**DOI:** 10.3389/fphar.2024.1342638

**Published:** 2024-03-21

**Authors:** Li Xiao, Hafsa Sunniya, Jingyi Li, Mohib Ullah Kakar, Rongji Dai, Bo Li

**Affiliations:** ^1^ Beijing Key Laboratory for Separation and Analysis in Biomedicine and Pharmaceuticals, School of Life Science, Beijing Institute of Technology, Beijing, China; ^2^ School of Medical Technology, Beijing Institute of Technology, Beijing, China; ^3^ School of Life Science, Beijing Institute of Technology, Beijing, China; ^4^ Advanced Research Institute of Multidisciplinary Science, Beijing Institute of Technology, Beijing, China

**Keywords:** *Bupleurum marginatum Wall.ex DC*, polysaccharides, anti-liver fibrosis, activity study, water extraction and alcohol precipitation

## Abstract

*Bupleurum marginatum Wall.ex DC* [Apiaceae] (BM)is widely grown in southwestern China, and the whole plant is used as Traditional Chinese Medicine (TCM). Polysaccharides are main natural products in lots of TCM and have been studied for their effects of reducing oxidative stress, anti-inflammation and immune regulation. Herein, we investigated the extraction techniques of *Bupleurum marginatum Wall.ex DC* polysaccharides (BMP), the identification of their key components, and their ability to inhibit liver fibrosis in both cellular and animal models. Component identification indicated that monosaccharides in BMP mainly consisted of glucose, galactose, mannose, rhamnose, arabinose, and xylose. *In vivo* analysis revealed that BMP provided significant protective effects on N-Nitroso dimethylamine (NDMA)-induced liver fibrosis rats through reducing hepatomegaly, reducing tissue inflammation, and reducing collagen deposition. BMP also improved the hepatobiliary system and liver metabolism in accord to reduce the serum levels of ALT, AST, ALP, r-GT, and TBIL. In addition, BMP could reduce the level of inflammation and fibrosis through inhibition of IL-1β and TGF-β1. Cellular studies showed that the BMP could provide therapeutic effects on lipopolysaccharide (LPS)-induced cellular fibrosis model, and could reduce the level of inflammation and fibrosis by decreasing the level of TGF-β1, IL-1β, and TNF-α. Our study demonstrated that BMP may provide a new therapy strategy of liver injury and liver fibrosis.

## 1 Introduction

Liver fibrosis usually occurs in the chronic process of trauma and healing, when the liver undergoes disease or encounters injury (Kukla, 2013), and extracellular matrix (ECM) deposition is the most distinctive feature of this pathological phenomenon (Trivella et al., 2020). After hepatocyte death, the cell contents release and interact with the Kupffer cell receptor that trigger the release of various cytokines including pro-inflammatory, fibrotic, and chemotactic factors. Hepatic stellate cells (HSCs) activate and proliferate rapidly under the stimulation of the above factors (Chan et al., 2020; Haaker et al., 2020). HSCs transformed into myofibroblasts together with hepatocytes. The differentiated HSCs then secrete a significant amount of ECM and deposit it extensively, which plays a crucial role in the development of liver fibrosis (Bao et al., 2021). Furthermore, normal hematopoietic stem cells contain abundant vitamin A lipid droplets in their cytoplasm. During liver tissue inflammation, HSCs can undergo similar differentiation, decrease cytoplasmic lipid droplets, enhance stem cell proliferation and protein synthesis, and secrete large amounts of ECM (Wang et al., 2020).

The current treatment for liver fibrosis primarily focuses on eliminating the factors that cause damage and reducing the underlying causes. This includes inhibiting the activation of HSCs, promoting the degradation of collagen deposits, reducing inflammation, and improving revascularization of liver tissue. Development of drugs to treat liver fibrosis have primarily been focused on small molecule compounds with anti-fibrotic activity (Shan et al., 2022). However, these drugs often have significant drawbacks such as high toxicity, low selectivity, poor therapeutic effects, and limited bioavailability (Tan et al., 2021). Some commonly used drugs in clinical practice include colchicine and interferon-γ, but their effectiveness in treating liver fibrosis is inconsistent and they often come with side effects such as nausea, diarrhea, fatigue, and headache (Rahman et al., 2022).

The treatment of liver-related diseases, including liver fibrosis, has been documented in traditional Chinese medicine since ancient times. According to TCM, liver fibrosis is primarily caused by qi deficiency and blood stasis (Li et al., 2020). The liver, as per TCM theory, stores blood, and any disturbance to the liver can result in blood stagnation and the formation of blood stasis along the meridians. Therefore, the key methods to treat liver diseases involving blood circulation promotion, removal of blood stasis, purging turbidity, and dredging collaterals. Yinchenhao decoction is commonly used to treat liver fibrosis associated with hepatobiliary damp-heat syndrome (Cai et al., 2019; Cai et al., 2019). It can alleviate liver injury by reducing inflammation, improving liver function, decreasing collagen content in liver tissue, and reducing oxidative stress. Fuzheng Huayu recipe, approved by the State Food and Drug Administration, is a therapeutic prescription for liver fibrosis that can inhibit the activation of HSCs and prevent liver tissue injury (Ping et al., 2024). TCM provides advantages in the treatment of liver disease, including its multi-target and multi-level approach.

Polysaccharides are biological macromolecules derived from natural products, known for their significant pharmacological effects (Yang et al., 2020). Known biological effects of polysaccharides include anti-tumor (Pan et al., 2017), anti-aging (Qian et al., 2018), anti-viral, anti-inflammatory, anti-oxidation (Song et al., 2013), anti-ulcer (Han et al., 2006), hypoglycemic (Ye et al., 2001), hypolipidemic (Dang et al., 2008), anti-coagulant (Fang et al., 2020), and immune function enhancing properties (Yuan et al., 2019). Polysaccharides from natural sources have shown promising therapeutic effects on various immune diseases and inflammatory damage diseases (Zeng et al., 2019). They are particularly advantageous as liver protective agents due to their ability to reduce oxidative stress, combat inflammation, and regulate the immune system (Xiao et al., 2018).

BM is widely distributed in Yunnan, Guizhou, Sichuan province in China, and it is classified under the liver and gallbladder meridians (Ashour et al., 2018). It is commonly used locally to treat conditions such as liver depression, qi stagnation, chest pain, and rib pain (Lei et al., 2019; Liu et al., 2021). The Chinese clinical preparation called “Baogan I” is utilized for liver disease treatment, with one of its main components being BM, which has demonstrated positive effects in combating liver fibrosis (Xia et al., 2021; Zhang et al., 2021). BM contains a variety of active ingredients (Luo et al., 2022). Our previous studies have isolated 25 monomeric compounds from BM, confirming their anti-liver fibrosis properties through network pharmacology, animal experiments, and cell experiments (Liu et al., 2018). However, there is limited research on the effective activity of polysaccharides derived from BM.

This study investigated the extraction techniques of polysaccharides from BM, identification of their key ingredients and examined their anti-liver fibrosis biological activities. The polysaccharide components were isolated and purified from the dried whole grass of BM using combination of water extraction, alcohol precipitation (Benvidi and Jahanbin, 2020), and column chromatography. Polysaccharides components were identified. The therapeutic and protective effects of BMP were evaluated in the rat liver fibrosis model induced by NDMA (Zhu et al., 2016). The study also examined the cytotoxicity of polysaccharides in the rat normal hepatocytes cells (BRL-3A) and further validated their anti-liver fibrosis activity in the LPS-induced cellular liver fibrosis model (Liang et al., 2016). Both *in vitro* and *in vivo* experiments confirmed the potential of natural polysaccharides extracted from BM as promising therapeutic drug to treat liver fibrosis.

## 2 Materials and methods

### 2.1 Materials

BM was purchased from Yunnan Jinfa Pharmaceutical Co., Ltd, and the whole plant was cut into the shape of 5–8 cm segments and dried. Bicinchoninic acid (BCA) protein assay kit was purchased from Beijing Solarbio Technology Co., Ltd. (Beijing, China). The TGF-β1, Interleukin-1β (IL-1β), tumor necrosis factor-α (TNF-α) and α-smooth muscle actin (α-SMA) enzyme-linked immunosorbent assay (ELISA) kits were purchased from Shanghai Guduo Biotechnology Co., Ltd. (Shanghai, China). Silymarin was purchased from Madaus GmbH, Germany. MTS kit was obtained from Promega (Beijing) Biotechnology Co., Ltd. (Beijing, China). Epigallocatechin gallate (EGCG) was purchased from Shanghai Macklin Biochemical Technology Co., Ltd.

### 2.2 Isolation and purification of polysaccharides from BM

#### 2.2.1 Extraction of polysaccharides from BM

The segmented BM was then crushed. A 2 L round bottom flask containing 50 g of BM powder was filled with 750 mL of petroleum ether at a ratio of 1:15 (mass/volume). After thorough mixing, the mixture was refluxed at 65°C for 1 h. The filtrate was discarded and the remaining petroleum ether was dried to obtain the defatted BM powder. This process was repeated 2–3 times. Next, 10 g of defatted BM powder was mixed with 350 mL of double-distilled water at a ratio of 1:35 (mass/volume). The mixture was thoroughly mixed and placed in an ultrasound generator, then heated at 50°C for approximately 1 h. The resulting suspension was transferred to a round bottom flask and heated on a reflux device with hot water at 90°C for 1–2 h. The extract was then filtered to remove any insoluble material. The resulting extract was rotated and evaporated to approximately 250 mL and papain was added to get a mass fraction of 0.2%. The mixture was then hydrolyzed at a temperature of 70°C for 1 h. After hydrolysis, the sample solution was heated to 100°C for 5–6 min to stop the enzyme reaction. Trichloroacetic acid was added to a concentration of 6% and the sample was deproteinized by shaking in a water bath at 70°C for 60 min. The precipitate was removed by centrifugation to eliminate denatured protein. The filtrate was collected and concentrated to 25 mL. Anhydrous ethanol was added to a concentration of 90% to precipitate the polysaccharides, which were left at 4°C for 12 h. The precipitate was collected by centrifugation at 4,000 rpm/min. Washing was performed using acetone, methanol, and anhydrous ethanol, and the remaining liquid was evaporated to obtain the BMP sample.

#### 2.2.2 Ultrafiltration membrane purification

Polysaccharides were separated according to their molecular weight. Before the experiment, the 300 mg of BMP dried and preserved in the above experiment was fully dissolved in 300 mL water. The sample solution was centrifuged by 2,000 rpm/min and prefiltered with 0.45 μ m aqueous membrane. The pre-filtered sample solution was placed in an ultrafiltration tank, the pressure of the nitrogen bottle was adjusted to 20psi (1psi = 6894.76 kPa), and ultrafiltration was carried out through 10 kDa and 30 kDa membranes at room temperature. The filtrate dripped after ultrafiltration was colorless and followed Molisch reaction to detect the presence of polysaccharides. The procedure was to prepare 1% α-naphthol in anhydrous ethanol as Molisch reagent. 1 mL of filtrate sample was taken at the outlet of ultrafiltration, added few drops of Molisch reagent, shaken and then mixed well. The tube was tilted and 1 mL of concentrated sulfuric acid was added slowly through the tube wall. The tube was then kept in a vertical position and the solution was divided in upper and lower portions to observe the purplish-red ring at the junction. If the solution contains sugar, the purplish-red ring appears in the solution. There was no Molisch reaction detected in the filtrate which indicated that the filtration was done, and the components inside and outside the membrane were recovered, dried and preserved after concentration.

#### 2.2.3 DEAE-52 column purification

The polysaccharides with molecular weight between 10 and 30 kDa were further purified and separated by DEAE-52 cellulose column. DEAE-52 cellulose column is mainly adsorbed and eluted by the principle of ion exchange. The reagents needed in this method are safe, non-toxic and have the potential of large-scale production and extraction. The dry powder of DEAE-52 ion exchanger was fully swollen until the pore was enlarged and the suspended impurities in the swelling process were discarded. After full swelling, they were alternately soaked in 0.5 mol/L HCl solution and 0.5 mol/L NaOH solution for more than 2 h, then filtered and rinsed repeatedly with double distilled water until pH was neutral. The suitable size chromatographic column was selected and fixed vertically on the iron platform, and the DEAE-52 column fillers treated with 20 mL or so were packed on the column and balanced with 5 volume double distilled water. The 120 mg of the dry sample to be separated was fully dissolved in a small amount of double distilled water, filtered by 0.45 μ m water-based filter membrane and pretreated by ultrasonic degassing. The sample was injected and the flow rate was adjusted to 1 mL/min. The 200 mL was eluted with double distilled water followed by 0.1, 0.2 and 0.3 mol/L NaCl solution in order while maintaining the flow rate. One tube was collected per 10 mL (20 min), and 20 tubes were collected from each component, with a total of 80 sticks. The OD value of each test tube was detected by phenol-sulfuric acid coloration method and the elution curve was drawn, and the fractions were merged according to the peak. Each combined component was passed through 500D membrane, and the AgCl reaction of the effluent showed that there was no precipitation, concentrated and dried and preserved. At the end of the experiment, the DEAE-52 ion exchanger was regenerated, then it was regenerated by alternately soaking in 0.5 mol/L acid-based solution for several times and washed to neutral in the same method as pretreatment, and finally stored in 20% ethanol at 4°C.

### 2.3 Analysis of BMP

#### 2.3.1 Physical and chemical properties determination

The total sugar content was determined by the phenol-sulfuric acid method. The determination of reducing sugar content was carried out using the sodium hydroxide, 3,5-dinitrosalicylic acid (DNS), potassium sodium tartrate method. The protein content was determined using the BCA method.

#### 2.3.2 Monosaccharide composition analysis

Sixteen monosaccharide standards were accurately weighed and configured as mixed standard samples as control. 5 mg of purified BMP sample was accurately weighed in an ampoule. 2 mL of 3 mol/L trifluoroacetic acid solution was added and the polysaccharides were hydrolyzed in an oven at 120°C for 3 h. After hydrolysis, the sample solution was transferred to a test tube, and nitrogen was used to evaporate it to dryness. The sample was re-dissolved with 5 mL of double-distilled water, and 50 μL was diluted 20 times and centrifuged at 12,000 rpm/min for 5 min. The supernatant was then removed. The ion chromatography was performed using a Dionex CarbopacTM PA20 (3*150 mm) column with mobile phases A. H_2_O, B. 15 mM NaOH, C. 15 mM NaOH & 100 mM NaOAC and monitored by an electrochemical detector.

#### 2.3.3 Ultraviolet characterization of polysaccharides

The purified BMP was accurately configured into a 1.0 mg/mL solution and pretreated using a 0.45 μM aqueous filter membrane. The total wavelength scan was performed at 190–400 nm using an Ultraviolet (UV) -visible spectrophotometer to observe the presence of absorption peaks at 260–280 nm to determine whether the polysaccharide fraction obtained from the separation and purification contained macromolecular impurities such as nucleic acids and proteins.

#### 2.3.4 Fourier transform infrared spectroscopy characterization of polysaccharides

Fourier Transform Infrared Spectroscopy (FT-IR) was performed using a Thermo Fisher Scientific Nicolet™ iS50 FT-IR spectrometer with dedicated accessories, integrated software, and an OMSNIC workstation with an operating range of 4,000–400 cm^−1^. The dried polysaccharide sample to be measured was placed directly on the sample stage and compacted with the probe to perform the range scan.

### 2.4 Animals feeding and model establishment

Male Wistar rats (SPF grade, 5–6 weeks old, weight 180 ± 10 g) were purchased from SiPeiFu (Beijing) Biotechnology Co., Ltd, certificate number: SCXK (Beijing) -2019-0010. The experiments were carried out in strict accordance with Decree No. 2 of the National Science and Technology Commission of the People’s Republic of China in 1988: Regulations on the Management of Laboratory Animals. All animal procedures such as housing, surgical procedures, pre-and post-operative care of the animals were performed in accordance with the ethical guidelines and approved by Beijing Institute of Technology (BIT-EC-SCXK2016-0006-M-2021065). All efforts were made to minimize animal suffering and to use as few animals as necessary.

Wistar rats were housed in isolated rearing cages with free access to water and food, and the bedding was changed every other day. The incubation room was set at 25°C ± 2°C temperature, 40%–60% humidity, 150 Pa pressurized air supply and 12 h cyclic light.

According to the dosing regimen (Supplementary Table S1), 36 male Wistar rats were arbitrarily divided into six groups; regular group (Control), model group (Model), positive drug group (PC), BMP low dose group (L-BMP), BMP medium dose group (M-BMP), and BMP high dose group (H-BMP) after 1 week of acclimatization. Every 7 days was an intervention cycle. The experiment was conducted for four cycles to model liver fibrosis caused by chronic liver injury and examine the therapeutic effects of BMP. Silymarin (Clichici et al., 2016), an extract of a Chinese herb, consists mainly of compounds such as flavonoids and lignans. It has been used as a marketed drug for many years in clinical anti-liver fibrosis applications, so Silymarin was used as a positive drug group, and a more optimal dose of 0.05 g/kg was selected for administration based on pre-laboratory work (Liu et al., 2018).

In the morning of the first three days of each cycle, 0.1 mL/100 g of 1% NDMA solution was injected intraperitoneally in the five groups, excluding the control group (Liu et al., 2017). The experimental rats in the different groups were fed with BMP or Silymarin for 4 weeks through oral administration from the first week of NDMN exposure. Body weights were recorded twice on days 1 and 4 of each cycle. After 4 cycles of feeding, the rats were fasted for 12 h. The rats were anesthetized by intraperitoneal injection of 10% chloral hydrate at a rate of 0.35 mL/100 g. Blood was extracted from the heart by cardioplegia and centrifuged at 3,500 r/min for 10 min after 3–4 h of resting. After blood sampling, an infusion syringe was used to perfuse pre-cooled saline through the heart, the perfusion was stopped until the liver turned white and there was no obvious blood clot. The liver and spleen were removed using surgical scissors, keeping the organs as intact as possible. Adherent tissues were stripped off, and excess blood was washed off in saline and gently patted dry. The liver index and spleen index (organ weight/body weight) were weighed and calculated. Part of the liver tissue was fixed in paraformaldehyde solution for histological analysis, and the remaining tissue was rapidly frozen at −80°C for long-term storage.

### 2.5 Histological analysis

The same position in the middle part of the left posterior lobe of the rat liver was selected, and the liver tissue of 0.5 cm × 0.5 cm was cut off with surgical scissors and transferred to paraformaldehyde fixed solution for 24 h. The fixed liver tissue was removed from the fixed solution and rinsed, embedded in paraffin, sliced and stained with H&E, and Sirius red.

### 2.6 Biochemical indexes

Use the fully automated biochemical analyzer AU480 from Japan’s Olympus company to detect serum biochemical indicators aspartate transaminase (AST), alanine transaminase (ALT), alkaline phosphatase (ALP), r-glutamyl transferase (r-GT), total bilirubin (TBIL), and glucose (GLU). The TGF-β1, IL-1β, TNF-α and α-SMA in liver tissues and cells were assayed using ELISA method as instructed by the manufacturer.

### 2.7 Cell culture

BRL-3A cells and HSCs-T6 were purchased from the Institute of Oncology, Cancer Hospital, Chinese Academy of Medical Sciences. The BRL-3A cells were cultured in DMEM containing 10% inactivated neonatal bovine serum and 1% penicillin-streptomycin in a 37°C, 5% CO_2_ incubator, and the HSCs-T6 were cultured in the same way as the BLR-3A cells.

### 2.8 *In vitro* toxicity experiments

Single-cell suspension was made from logarithmic growth phase BRL-3A cells, and the cell concentration was diluted to 3 × 10^4^/mL and added to a 96-well plate at 100 μL per well to reach 3000 cells/well. The cells were placed back into the cell culture incubator for 24 h and cultured until the cells were attached to the wall in a good condition.

The dried polysaccharide extracts were completely dissolved and diluted to different concentrations using a complete medium. The 96-well plate was washed twice with PBS, 100 μL/well of a medium solution containing different concentrations of polysaccharide extracts was added, and three replicate wells were set up. The 96-well plates were labeled and disinfected with alcohol and incubated for 24 h. The cultural conditions remained unchanged.

The MTS method was used to test cell proliferation and survival, based on the proportional relationship between the different absorbance values at 490 nm.

### 2.9 Model establishment and cell viability assay

Normal state HSCs-T6 in log phase were treated to obtain suspension and their cell concentration was diluted to 10^4^/mL, and 100 μL per well was added to a 96-well plate to 1000 cells/well. The cells were incubated normally for 24 h until the cells were adherent in prime condition. LPS solution was prepared in gradient concentrations of 0.1, 0.5, 1, 2, 5 and 10 μg/mL, and 4 replicates of each concentration were added sequentially in 96-well plates, and 4 additional wells were set as blank controls. Normal incubation was performed for 24 h. The culture conditions were unchanged. The morphological changes of HSCs-T6 were observed and the value-added rate of HST-T6 cells was measured by MTS method.

Dried BMP was completely dissolved and diluted to different concentrations using cell culture medium. EGCG was used as a positive drug in cell experiments. The existing cell culture medium in the 96-well plate was discarded, washed with PBS and 100 μL/well of the configured medium solution containing BMP or EGCG was added. Four replicate wells were set up with the same culture conditions and incubated normally for 24 h. The cell survival rate was measured by the MTS method to investigate the inhibitory effect of BMP.

### 2.10 Statistical analysis

Statistical analysis was performed using GraphPad Prism software (GraphPad Software, USA). All error bars were represented as means ± SD, differences detection index between the treated groups and control group were determined via one-way analysis of variance (ANOVA). *p* < 0.05 was considered significantly different.

## 3 Results

### 3.1 BMP-M obtained by ultrafiltration membrane purification

Polysaccharides contain a large number of polar groups and their molecular weight is generally large. With the increase of temperature, the solubility of polysaccharides in water increases. Thus, polysaccharides could be extracted from natural plants by hot water extraction (Wu et al., 2020). With the increase of alcohol concentration in the extract, the solubility of polysaccharides decreased and the purified polysaccharide extract was gradually precipitated. Based on the above principles, the BMP sample was obtained by water extraction and alcohol precipitation. After the BMP sample was filtered and purified by 10 kDa ultrafiltration membrane, the sample in the ultrafiltration tank was recovered, producing 269 mg with the recovery rate of 89.7%. 10 kDa ultrafiltration membrane can effectively remove some small molecular compounds in the sample solution and reduce their impact on subsequent monosaccharides identification. After filtration and purification by 30 kDa ultrafiltration membrane, polysaccharide sample (BMP-M) 57 mg with a molecular weight of 10–30 kDa was obtained, accounting for about 19.0% of BMP. The molecular weight of this part of polysaccharides is relatively uniform, which is used for subsequent identification and research. In addition, the recovery of macromolecular polysaccharides in the ultrafiltration tank, including 187 mg, accounts for about 62.3% of BMP.

### 3.2 BMP-D obtained by DEAE-52 column purification

**FIGURE 1:**
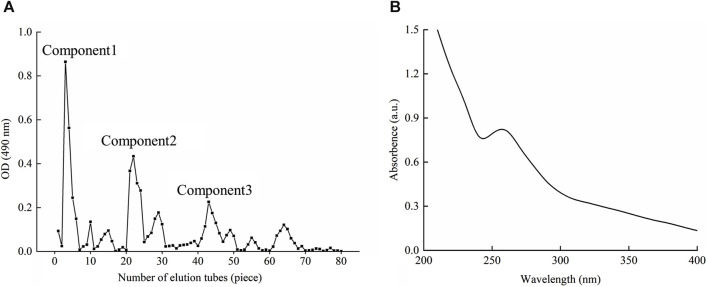
**(A)** DEAE-52 column elution curve. **(B)** UV scanning spectrum of the BMP-D.

According to the chromogenic absorption value of sulfuric acid-phenol and the number of collecting tubes, the elution curve was drawn as shown in Figure 1A. As could be seen from the diagram, the elution peak appeared soon after the sample was eluted with double distilled water, and the component was basically collected in the first 8 test tubes, which was named component 1. With the extension of elution time, two small elution peaks appeared, but their concentrations were low and did not get to the significant elution peaks. When the mobile phase was changed to 0.1 and 0.2 mol/L NaCl, there were obvious elution peaks, and the components were collected and named as components 2 and 3. Similarly, the concentration of polysaccharides obtained by elution was lower over time. After changing the mobile phase to 0.3 mol/L NaCl, there was no obvious elution peak, so it was speculated that most of the polysaccharides after exchange have been eluted and separated. Components 1, 2 and 3 were combined and weighed after drying, and the recoveries were 43.14%, 17.43% and 13.80%, respectively. Under this elution condition, the elution peak of component 1 was symmetrical, the peak was the highest and the content was in the highest amount. The component 1 was named BMP-D and its component was identified later.

### 3.3 Analysis of BMP-D

BMP-D was first hydrolyzed using concentrated sulfuric acid, exposing the primary hydroxyl groups on the sugar chain or at both ends, and rapidly form 2-furaldehyde derivatives after oxidative dehydration with concentrated sulfuric acid. 2-Furaldehyde derivatives combined with phenol to form an orange-yellow substance with the maximum absorbance value at 490 nm. The BMP-D sugar content was determined as 58.29% with glucose as the standard using the phenol-sulfuric acid colorimetric method.

The polysaccharides were broken down and hydrolyzed in high temperature water bath to produce reducing sugar, which could produce 3-amino-5-nitrosalicylic acid after combining with 3,5-DNS under alkaline condition. The substance was brownish-red in color with maximum absorbance at 550 nm. The content of reducing sugar in BMP-D was measured to be 6.42%.

The content of protein in BMP-D was 18%. This could be due to the fact that some proteins formed complexes with polysaccharides by covalent bonds, which were relatively stable and might be embedded in the complex polysaccharide. Referred in the literature, the polysaccharide-protein complex provided biological activities including antioxidation, anti-inflammation and thermal stability, and it was difficult to be removed by conventional methods (Yu et al., 2018).

**FIGURE 2:**
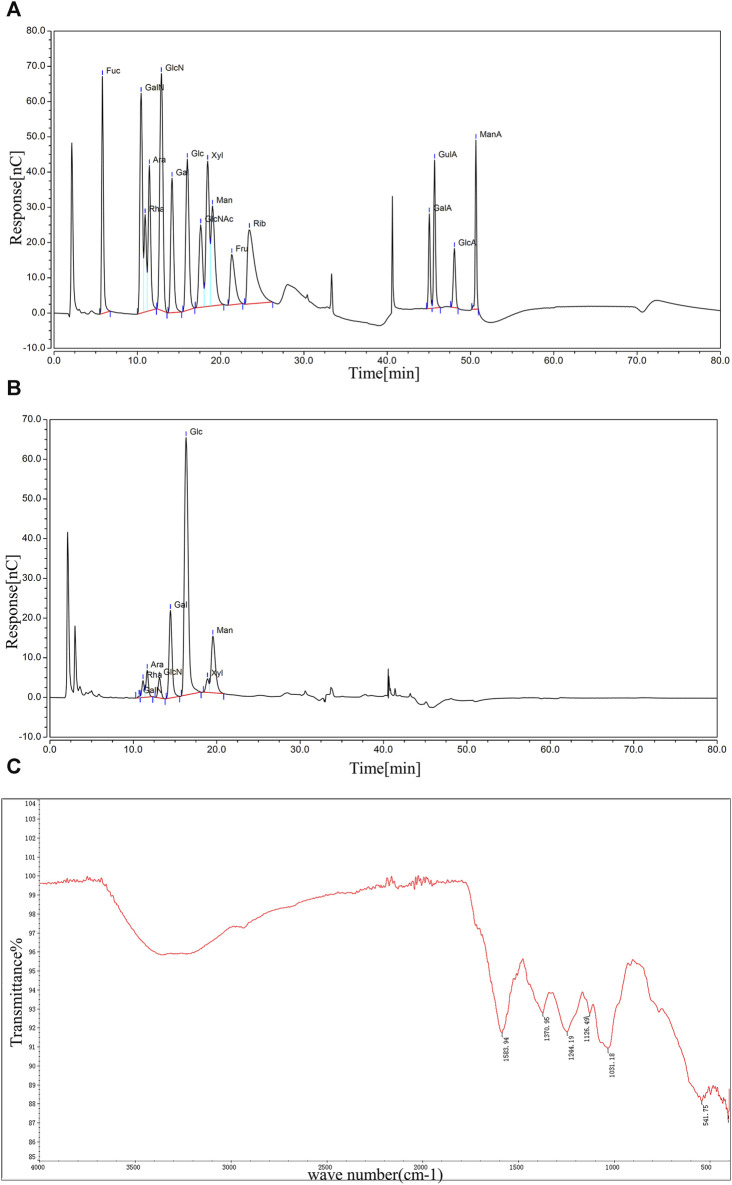
**(A)** Ion chromatogram of mixed standard sample. **(B)** Ion chromatogram of BMP-D sample. **(C)** FT-IR scanning spectrum of BMP-D sample.

Saccharide molecules have the property of being able to assume an ionized state in strong alkaline solutions. The polysaccharide molecules can be fully acid-hydrolyzed to monosaccharides, which can be separated by the principle of different ion-exchange interactions of different types of monosaccharides, and detected by electrochemical methods. Figure 2A showed the ion chromatograms of 16 monosaccharides mixed standard samples, and it could be observed that each monosaccharide embodied independent and obvious peaks with different peak times and peak areas (Supplementary Table S2). The ion chromatograms of the BMP-D samples were shown in Figure 2B, compared with the mixed standard chromatogram, and it could be seen that BMP-D mainly consisted of rhamnose, arabinose, galactose, glucose, mannose, and contained a small amount of xylose, glucosamine hydrochloride, and amino galactose hydrochloride. The peak times and peak areas of the monosaccharides were integrated and counted, and the percentage of each monosaccharide was further calculated, which was shown in Table 1. From the table, it could be seen that the main monosaccharide components in the BMP-D were glucose (53.81%), galactose (17.97%), mannose (15.56%), rhamnose (4.77%), and arabinose (3.56%).

**TABLE 1:** Monosaccharide ratio in BMP-D.

Order number	Name	Peak time (min)	Monosaccharide mass (μg/mg)	Percentage content (%)
1	Galactosamine hydrochloride	10.692	0.34	0.1
2	Rhamnose	11.159	15.73	4.77
3	Arabinose	11.659	11.74	3.56
4	Glucosamine hydrochloride	13.134	6.73	2.04
5	Galactose	14.45	59.27	17.97
6	Glucose	16.325	177.49	53.81
7	Xylose	18.892	7.21	2.19
8	Mannose	19.542	51.34	15.56

The BMP-D was scanned at full wavelength in the range of 200–400 nm, as shown in Figure 1B. According to the graph, it could be seen that there was a small absorption peak at 260 nm, which indicated that there was a certain amount of protein in the polysaccharide, which should be covalent bond, so it was not removed in the deproteinization step. There was no obvious absorption peak at 280 nm, which indicated that the polysaccharide extract had no obvious nucleic acid impurities after purification.

FT-IR can effectively predict and identify polysaccharide macromolecules, and the FT-IR spectrum of BMP-D is shown in Figure 2C. The absorption band at 3,200–3,700 cm^−1^ indicated the presence of the O-H stretching vibration of the hydroxyl group. A weaker characteristic absorption band at 2,930 cm^−1^ indicated the presence of C-H stretching. A clear absorption peak existed at 1,583 cm^−1^, indicating a possible C-O stretching vibration of the carbonyl group. The absorption peaks at 1,370 cm^−1^ and 1,244 cm^−1^ corresponded to C-H bending and C-C stretching vibrations. The faint absorption peak at 1,126 cm^−1^ could be other vibrations of ketones. In addition, the distinct characteristic absorption band at 1,031 cm^−1^ indicated the presence of a CO-O-CO bond in the polysaccharide extracts. The infrared spectrum absorption peak showed that there was a distinct polysaccharide structure in the extracted component.

### 3.4 BMP decreased liver index and increased body weight in rats with NDMN-induced liver fibrosis

After initiation of the NDMN injections, the survival rate and body weight of rats were measured, and their condition was continuously observed. The rats in the normal group exhibited high energy levels, had white and smooth fur, good appetite, and consumed a high amount of water. On the other hand, the rats in the model group displayed loss of appetite, decreased mental and physical strength, yellowish and dry fur, and an emaciated body. After administering BMP with low and medium doses or PC through gavage, the above symptoms were significantly reduced, the rats showed livelier behavior and their fur regained its glossiness. However, in the H-BMP group, the situation did not improve significantly.

**FIGURE 3:**
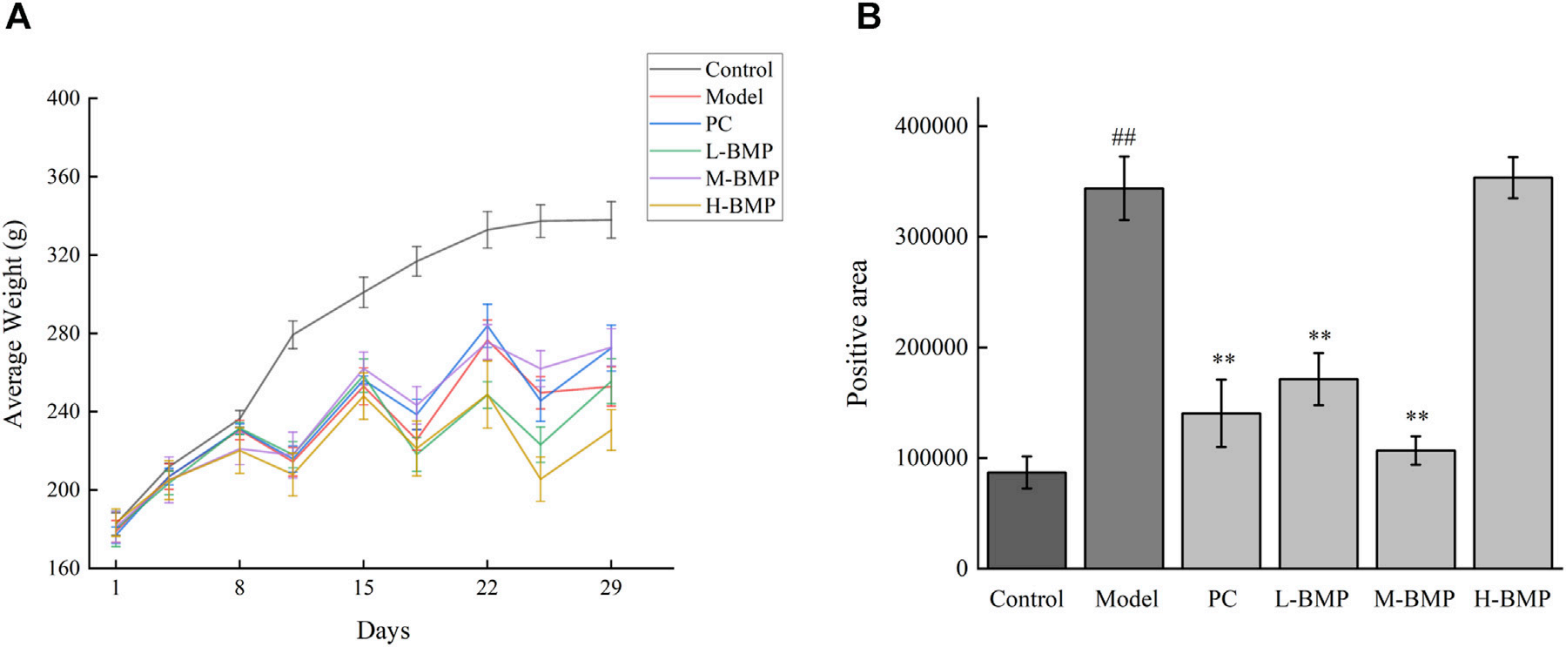
**(A)** Changes in body weight of rats over time. **(B)** Sirius red stained section positive area. ^##^ *p* < 0.01, versus control group. **p < 0.01, versus model group.

The changes in rats’ body weight before and after the feeding stage are shown in Table 2. As shown in the table, the body weights of the rats were all closer to each other before the intervention, whereas their weights after NDMA modeling were all significantly reduced. This might be attributed to the fact that liver injury affected the physiological state of the normal rats, resulting in dietary deficiencies and metabolic disorders. Among them, the body weights of rats in the PC group and the M-BMP rebounded, which reflected the protective and therapeutic effects of the drug. The modeling effects of the intraperitoneal injection of NDMA were shown in Figure 3A. The body weight of the rats increased slowly in the first week, and then decreased in the first three days of the second, third, and fourth weeks, and then regained the body weight in the last four days, which indicated that the NDMA-induced modeling affected the rats’ normal feeding, digestion and metabolism.

**TABLE 2:** Effects of BMP on weight of liver fibrosis rats.

Group	Dose (mg kg^−1^ d^−1^)	Weight before intervention (g)	Weight after intervention (g)
Control	-	182.7 ± 5.7	338.0 ± 9.3
Model	-	178.9 ± 5.6	252.9 ± 10.0^##^
PC	50	177.0 ± 4.2	272.5 ± 11.7*
L-BMP	100	180.3 ± 9.1	255.6 ± 11.5
M-BMP	200	181.2 ± 8.0	272.9 ± 9.5*
H-BMP	400	183.4 ± 7.0	230.7 ± 10.4**

Values represented as mean ± SD. ^##^ *p* < 0.01, versus control group. *p < 0.05, **p < 0.01, versus model group.

The liver index indicates the size of energy consumption and whether the morphology of the rat changes (Koyama et al., 2016). The spleen index reflects the body immunity of rats, and its weight and function correlate with the immune activity (Lai and Afdhal, 2019). The liver and spleen indexes of the rats were accurately weighed and calculated after autopsy. As shown in Table 3, after NDMA induction, the liver index of the five groups increased, which might be due to liver lesions, swelling and inflammation caused by drug administration. It was observed that the liver index dropped significantly to nearly normal levels with the treatment of the positive drug Silymarin and the medium dose of BMP. The M-BMP polysaccharides were slightly more effective than the positive drug. This indicated that this medium BMP dose could treat the liver’s enlargement and reduce the lesion response. The morphological changes in rat liver were evident. The liver of the normal group rats was rosy and smooth, full of tissue, with no noticeable texture and heavy quality. The liver of the model group showed atrophy and dryness, slightly hard tissue, filiform texture, and yellowish and dull color. The condition of the liver after drug treatment was relieved, more rosy color, softer texture, and less obvious texture.

**TABLE 3:** Effects of BMP on liver and spleen indexes in liver fibrosis rats.

Group	Dose (mg kg^−1^ d^−1^)	Liver weight (g)	Liver index (g)	Spleen weight (g)	Spleen index (g)
Control	-	9.80 ± 0.40	2.90 ± 0.06	0.77 ± 0.15	0.23 ± 0.04
Model	-	9.00 ± 0.86	3.55 ± 0.24^##^	1.25 ± 0.18^##^	0.49 ± 0.08^##^
PC	50	8.21 ± 0.39	3.02 ± 0.01**	1.18 ± 0.27	0.43 ± 0.10
L-BMP	100	8.70 ± 1.24	3.40 ± 0.38	1.31 ± 0.28	0.51 ± 0.10
M-BMP	200	7.91 ± 0.92	2.90 ± 0.31**	146 ± 0.39	0.53 ± 0.13
H-BMP	400	7.68 ± 0.93*	3.33 ± 0.33	1.33 ± 0.49	0.58 ± 0.22

Values represented as mean ± SD. ^#^ *p* < 0.05, ^##^ *p* < 0.01, versus control group. *p < 0.05, **p < 0.01, versus model group.

### 3.5 BMP ameliorated liver injury

**FIGURE 4:**
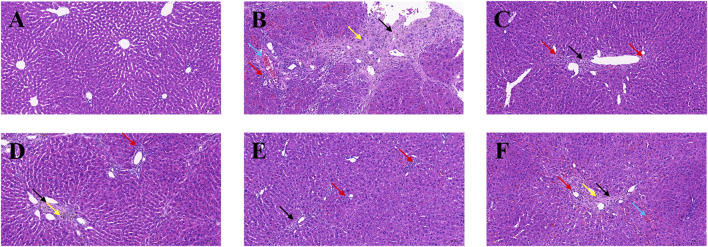
Effects of BMP on the histopathological changes of rat liver tissue. H&E staining (20.0×). **(A)** Control group, **(B)** Model group, **(C)** PC group, **(D)** L-BMP group, **(E)** M-BMP group, **(F)** H-BMP group.

To evaluate histological changes in the liver, H&E and Sirius red stained tissue sections from each group were assessed. The liver tissue was fixed and sectioned, stained with H&E. As shown in Figure 4, in the normal group (Figure 4A), the lobular structure of the liver was apparent, and the cell cords were arranged in an orderly manner. No pathology such as dilatation or stenosis of the liver sinusoids was observed. Inflammatory cell infiltration could be eliminated. Compared with the normal group, the liver lobule structure of the modeled rats (Figure 4B) was more disorganized, with overall abnormal tissue structure and severe fibrosis, with multiple foci of hepatocyte necrosis (black arrows), loss of normal structure, and fragmentation and disappearance of nuclei. Pronounced inflammatory cell infiltration (red arrows) and hemosiderin deposition (yellow arrows) were observed in the liver lobules and confluent areas, with an edema-like, with mild stasis in the veins and tissue spaces (blue arrows). Large lipid vacuoles appeared, forming coarse fibrous septa, and this section indicated successful modeling of liver fibrosis.

In contrast, the liver lobules in each administration group (Figures 4C–F) restored a clearer and more orderly structure and more regular distribution of hepatocytes. However, the overall tissue was mildly fibrotic, with localized connective tissue hyperplasia (black arrows), partial inflammatory infiltration (red arrows), and slight hemosiderin deposition (yellow arrows) still observed in the confluent area. However, the extent was significantly less than that of the modeling group. The extent was significantly reduced compared with the model group. Among them, the low and medium doses of BMP and the positive drug Silymarin group were more effective in maintaining the typical morphology of liver lobules and improving the fibrotic morphology, in which the lipid vacuoles and fibrous septa were also reduced, and the pathological state was significantly remarkably reduced.

**FIGURE 5:**
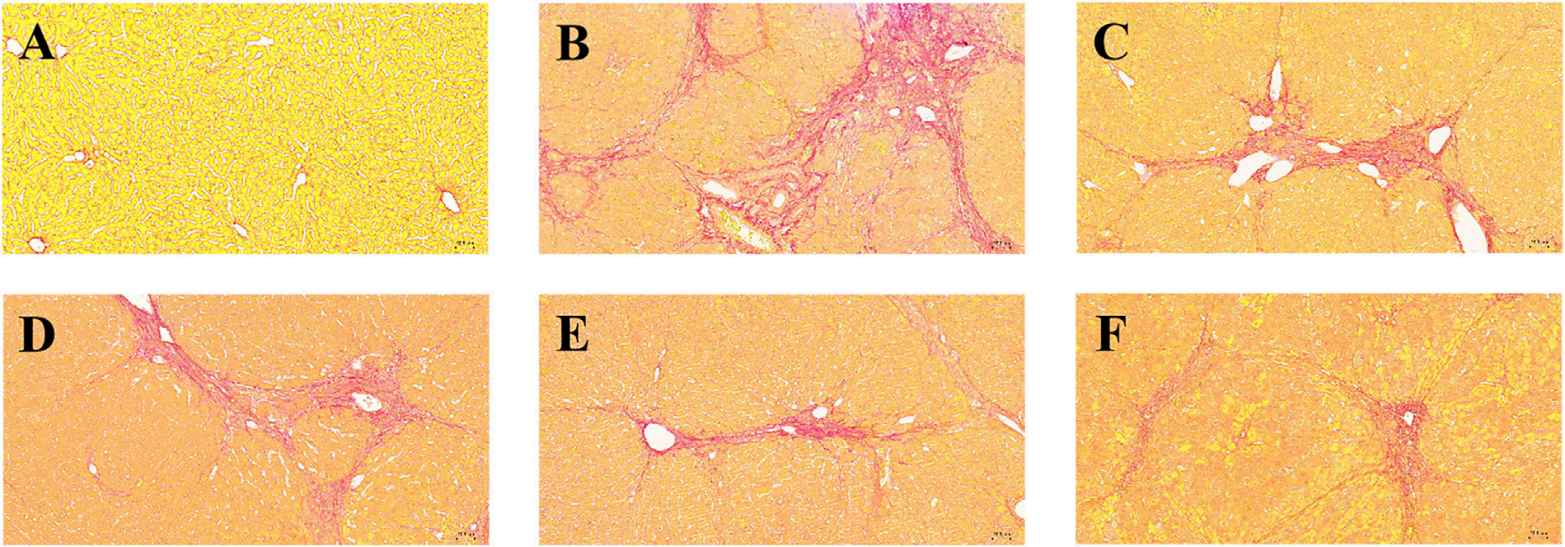
Effects of BMP on the histopathological changes of rat liver tissue. Sirius red staining (10.0×). **(A)** Control group, **(B)** Model group, **(C)** PC group, **(D)** L-BMP group, **(E)** M-BMP group, **(F)** H-BMP group.

Collagen deposition status, extent, and distribution in liver fibrotic tissue could visualized using Sirius red staining. The background is yellow under the light microscope, where type I collagen deposition is red. It could be seen that the normal group (Figure 5A) had an overall yellow color of the liver tissue, with only a light red color around some of the cavities, indicating that the collagen in the tissue was in the minority. In the model group (Figure 5B), it could be seen that the overall liver tissue background was orange-red, indicating that collagen deposition existed in the interstitial space of liver cells. There were apparent red collagen thick fibers around the interstitial space of the liver sinusoids in a ring-like distribution. The collagen in the portal canal area of the liver was distributed in a radioactive stellate shape with noticeable crimson color. In addition, large collagen thick fibers were also distributed in a sparse network in the tissue, and there were areas of fibrous septa and nodules. After the treatment with positive drug Silymarin and BMP (Figures 5C–F), it could be seen that the crimson coarse fiber areas were significantly reduced. The collagen deposition phenomenon was improved, and the still existing red areas were mainly concentrated around the portal area. The interstitial space was in a ring or strip-like distribution, in which the fibrous septa, fibrous bridging, and nodules were less reflected, which reflected that BMP provided specific therapeutic and protective effects on liver fibrosis.

**FIGURE 6:**
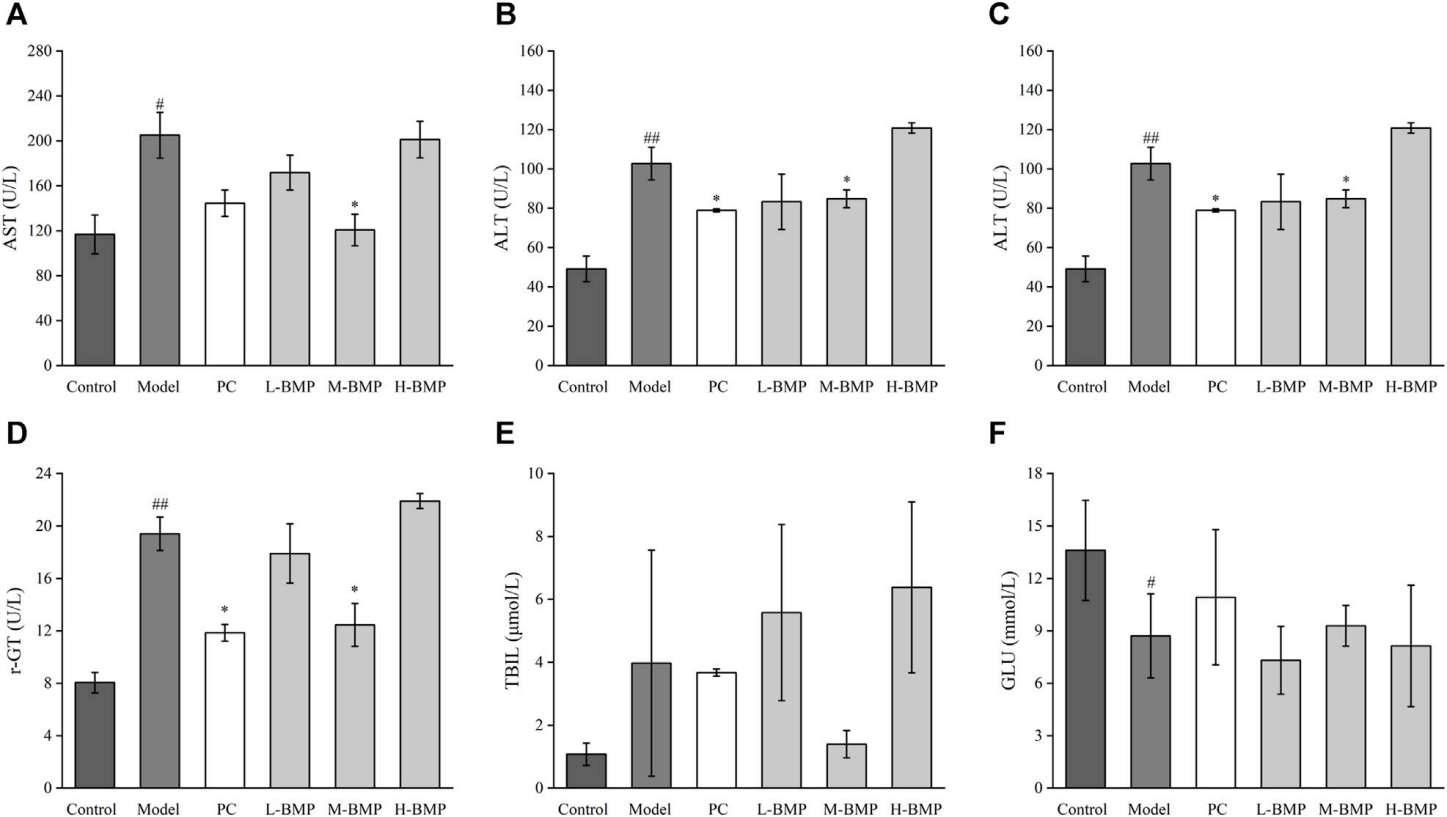
Effects of BMP on serum biochemical indexes in rats. **(A)** AST, **(B)** ALT, **(C)** ALP, **(D)** r-GT, **(E)** TBIL, **(F)** GLU. ^#^ *p* < 0.05, ^##^ *p* < 0.01, versus control group. *p < 0.05, **p < 0.01, versus model group.

The Sirius red stained images were analyzed by Image pro-plus 6.0 software, the blank areas in the field of view were minimized, and a consistent background light was selected to avoid errors during processing. As shown in Figure 3B, the positive area of collagen deposition in the model group was more than three times that of the normal group compared with the normal group, indicating that NDMA modeling led to a large amount of ECM secretion and severe collagen deposition in the tissue, which was a typical feature of liver fibrosis. This indicated that the modeling was effective. Treatment with the positive drug Silymarin and low and medium doses of BMP could effectively control the collagen deposition in the tissues, with the medium dose of BMP treatment being the most effective and able to control the positive collagen area to only slightly higher than that of the normal group. However, the therapeutic effect of the high dose of BMP was not reflected. Combined with the above analysis, too high dose of BMP can aggravate the burden of liver metabolism, which may lead to damage to intrahepatic cells, resulting in the release of intracellular collagen and distressed metabolism.

### 3.6 BMP restored serum biochemical indexes of liver function and inhibited the expression of fibrosis factors and inflammatory factors

The biochemical indexes of liver function in rat serum were detected and used as a standard of the degree of liver function damage. The results are shown in Figure 6. Compared with the control group, the levels of AST, ALT, ALP, r-GT, and TBIL in the serum of rats after NDMA induction were significantly increased, indicating that the liver tissue cells were damaged, and inflammation, metabolic abnormalities, and jaundice were observed. After treatment with PC, L-BMP or M-BMP, the AST levels were downregulated, with the AST levels in the M-BMP group reaching normal, indicating that BMP treatment at that dose was the most effective and could effectively reduce hepatocyte damage. The serum ALT levels were also downregulated after PC, L-BMP or M-BMP treatment, which reduced the inflammation and damage in the tissues. The serum ALP and r-GT indexes showed that the PC and M-BMP had better therapeutic effects, which could improve the metabolic capacity of the liver and maintain the regular operation of the hepatobiliary system. The TBIL level showed that only M-BMP had a better improvement function, which indicated that it could reduce the phenomenon of jaundice to a certain extent. The higher GLU content in the serum of the rats in the control group indicated that the liver metabolism was vigorous, and the GLU content could be significantly reduced after NDMA modeling. This indicated that the corresponding damage was caused due to the liver function metabolism. After treatment with PC and M-BMP, GLU levels in serum rebounded, indicating their ability to treat and protect liver metabolism-related functions.

**FIGURE 7:**
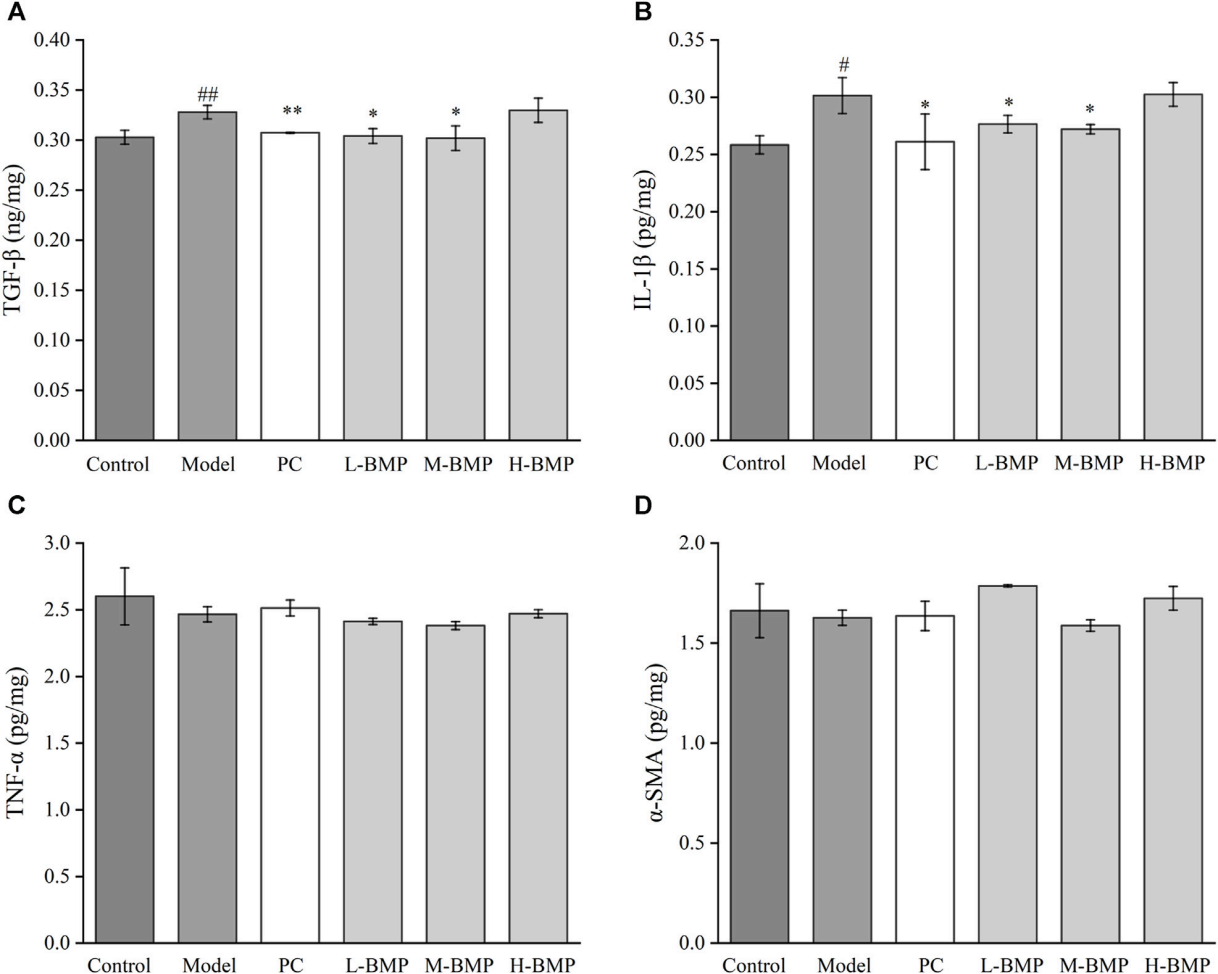
Effect of BMP on biochemical indexes of rat liver tissue **(A)** TGF-β1, **(B)** IL-1β, **(C)** TNF-α, **(D)** α-SMA. ^#^ *p* < 0.05, ^##^ *p* < 0.01, versus control group. *p < 0.05, **p < 0.01, versus model group.

Compared with the normal group, TGF-β1 and IL-1β were increased after modeling (Figures 7A, B), indicating a significant inflammatory and fibrotic response in the NDMA-induced rats. After treatment with PC, L-BMP or M-BMP, the levels of these factors decreased. It revealed that BMP could be anti-fibrotic by inhibiting fibrosis-related targets in liver tissues and reducing tissue inflammation. TNF-α and α-SMA (Figures 7C, D) did not show significant changes under this model. After NDMA modeling, the rat liver was in a moderate fibrotic state, with fibrotic filaments and slight sclerosis, ECM deposition, protein entanglement and cross-linking, and homogenization and conventional protein characterization methods were unable to release the various inflammatory factors within the liver fully (Pinzani, 2015). In addition, to further validate the changes of these cytokines, HSCs-T6 activated *in vitro* liver fibrosis model was designed.

### 3.7 Evaluation of cytotoxicity of BMP

**FIGURE 8:**
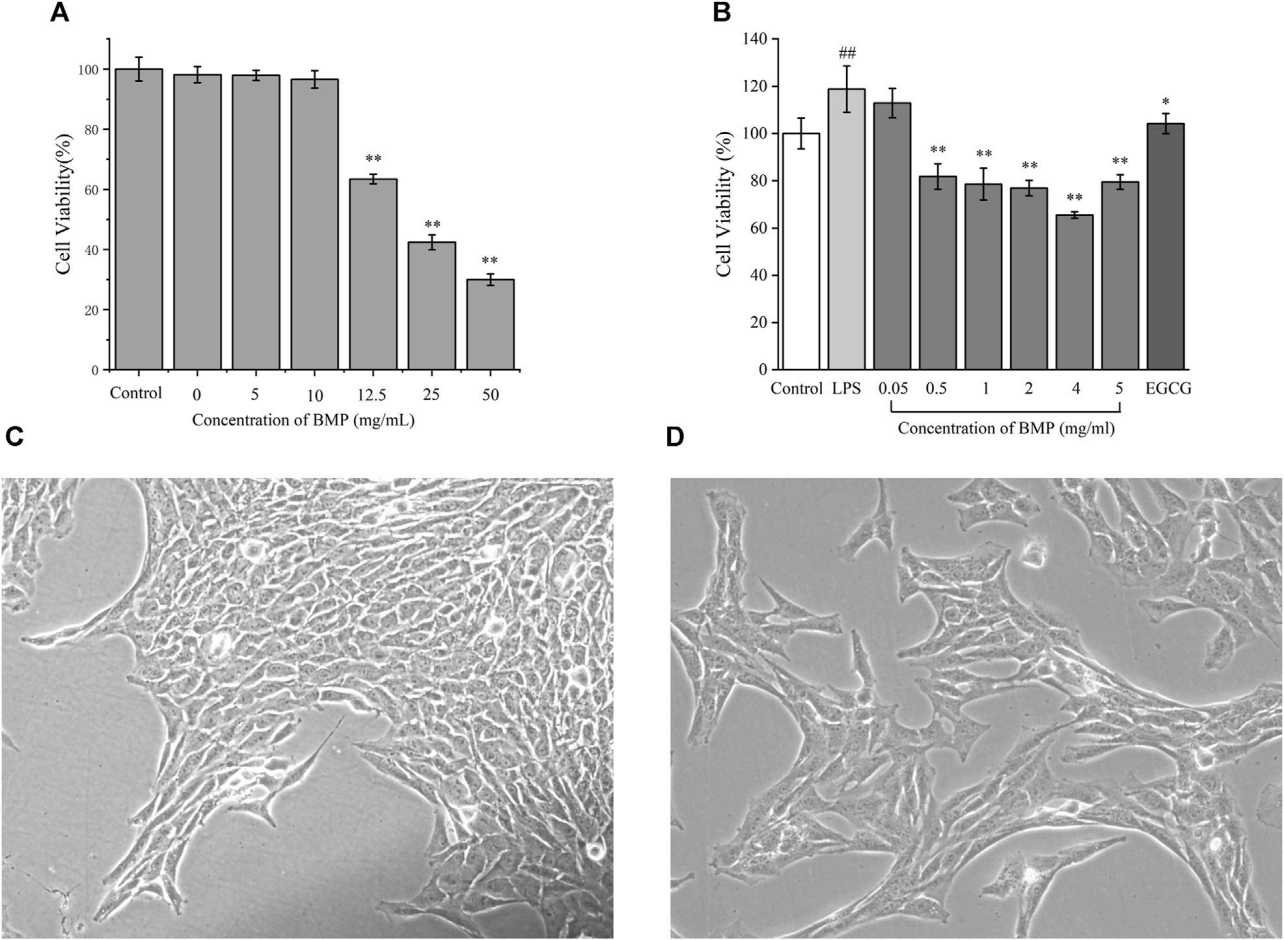
**(A)** Effects of different concentrations of BMP on the survival rate of BRL-3A cells. **(B)** Inhibitory effect of BMP on LPS-activated HSCs-T6 proliferation. Micrographs of HSCs-T6 **(C)** static, **(D)** LPS activation. ^#^ *p* < 0.05, ^##^ *p* < 0.01, versus control group. *p < 0.05, **p < 0.01, versus model group.

The toxicity assay of BMP on normal rat hepatocytes were carried out and the result is shown in Figure 8A. The effect of polysaccharides on the survival of hepatocytes was low when the polysaccharide dose was below 10 mg/mL, and there was a significant dose-dependent inhibition of cell growth when the dose was above 12.5 mg/mL. The high concentration of polysaccharides caused a considerable metabolic burden on hepatocytes, producing metabolites such as fat and a significant accumulation of some low toxicity groups on polysaccharides, which had adverse effects on cells. In addition, high concentrations of water-soluble polysaccharides may affect the osmotic pressure of the cellular environment, resulting in an adverse effect on the cellular growth state.

### 3.8 BMP inhibited LPS-activated HSCs-T6 proliferation

LPS was used to activate and proliferate HSCs-T6 in the resting state in order to set up the cellular model of liver fibrosis. The activated HSCs-T6 had rapid proliferation, morphological contraction, and rapid promotion of ECM secretion. Based on the pre-experiment results (Supplementary Figure S1), the best activation effect was achieved when the LPS concentration was 1 μg/mL. Figure 8C showed the inactivated steady-state HSCs-T6 grown against the wall and were epithelial-like with irregular trilateral or polygonal cytosol. Figure 8D showed that HSCs-T6 were activated upon addition of 1 μg/mL LPS concentration. The cells were enlarged. The morphology was laterally contracted and longitudinally elongated, converging to fibrous shape. The induction of differentiation was turned on.

The inhibitory effect of BMP on activation of HSCs-T6 proliferation was shown in Figure 8B. From left to right, the control group, LPS modeling group, and BMP concentrations of 0.05, 0.5, 1, 2, 4, and 5 mg/mL with the positive drug EGCG at a concentration of 10 μM, respectively. Experiments showed that BMP concentrations higher than 0.05 mg/mL showed better inhibition of proliferation of HSCs-T6, among which the best dose was 4 mg/mL.

### 3.9 BMP decreased the levels of fibrosis factors and inflammatory factors expressed by HSCs-T6

**FIGURE 9:**
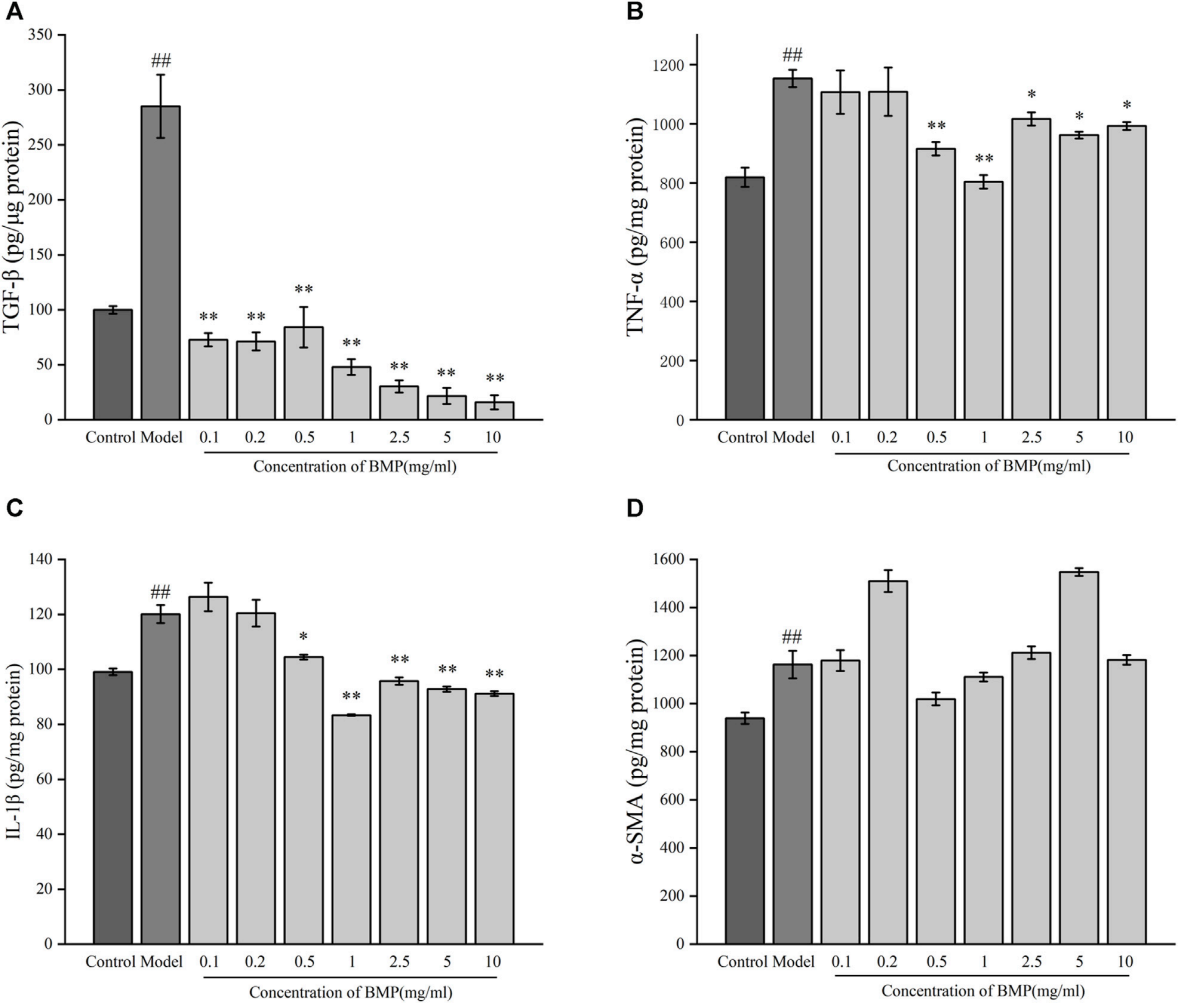
Effects of BMP on biochemical indexes of LPS-activated HSCs-T6. **(A)** TGF-β1, **(B)** TNF-α, **(C)** IL-1β, **(D)** α-SMA. ^##^ *p* < 0.01, versus control group. *p < 0.05, **p < 0.01, versus model group.

TGF-β1 is one of the most critical cytokines in liver fibrosis, involving multiple upstream and downstream pathways related to the process of liver fibrosis and was also involved in the release of ECM (Dai, 2022). As shown in Figure 9A, the content of TGF-β1 in the model group increased twice as much as that in the normal group. In each administration group, BMP was able to significantly inhibit the level of TGF-β1 in activated HSCs-T6. Among them, this factor could decrease to the normal group level when the concentration of BMP was 0.1–0.5 mg/mL, and the inhibitory effect was increased as the administered dosage increased when the concentration was higher than 1 mg/mL. The BMP did not produce significant toxicity to normal hepatocytes at 10 mg/mL. Therefore, it was speculated that BMP could inhibit liver fibrosis by reducing the level of TGF-β1.

TNF-α plays an important role in the regulation of collagen deposition and degradation in the process of liver injury and repair (Campana and Iredale, 2017). As shown in Figure 9B, after LPS induction, the content of TNF- α in activated HSCs-T6 increased by 40% compared with the steady state, indicating that liver fibrosis would lead to the release of TNF-α. Compared with the model group, BMP could notably significantly reduce the content of TNF-α when the concentration was 0.5 and 1 mg/mL. The inhibitory effect of BMP at 1 mg/mL was better. When the concentration was higher than 2.5 mg/mL, it did not show significant inhibition, which might play a reverse action in the regulatory mechanism of repairing an injury. Generally speaking, BMP at low and medium doses played a role in the treatment of liver fibrosis by down-regulating TNF-α.

The cytokine IL-1β is an essential factor released during liver injury and can induce an extensive synthesis and secretion of other inflammatory, oxidative stress-related cytokines (Dhar et al., 2020). As shown in Figure 9C, in the LPS-activated liver fibrosis cell model, the content of IL-1β increased by 20% compared with the normal group, indicating an increase in inflammation. BMP exhibited a substantially inhibitory effect on IL-1β content when the BMP concentration was higher than 0.5 mg/mL, whereas the inhibition was more effective when its level was 1 mg/mL. These results indicated that BMP played important role in reducing inflammation levels and slowing down fibrosis.

α-SMA is one of the main components of the extracellular machinery expressed and released during the onset of liver fibrosis. α-SMA provides the effect of promoting HSCs differentiation and releasing ECM, thus promoting collagen deposition and exacerbating the process of liver fibrosis (Luangmonkong et al., 2023). As shown in Figure 9D, it can be seen that after cellular liver fibrosis activation, the level of α-SMA elevated. However, there was no obvious regularity of α-SMA content after administration of any drugs.

## 4 Discussion

The pathological condition of hepatic fibrosis is partially reversible, especially when the excessive deposition of ECM in the liver is still in a mild state in the early stage, the pathological injury is likely to improve and recover. It is very important to locate the liver fibrosis and treat it early before the liver lesion develops to liver cirrhosis or liver cancer. However, at present, there is no widely accepted effective anti-fibrosis drug, so it is very important to carry out corresponding research in this direction.

Herein, polysaccharides from BM were extracted to study the therapeutic effect of BMP on liver fibrosis. BMP were extracted by water extraction and alcohol precipitation, and the polysaccharides were purified and identified. In addition, the pathological model of hepatic fibrosis *in vivo* was established by intraperitoneal injection of NDMA into Wistar rats, and the pathological model of hepatic fibrosis *in vitro* was established by activating HSCs-T6 with LPS. The therapeutic effect of BMP on hepatic fibrosis was studied. The results are summarized as follows:

Polysaccharides were extracted from BM. The small molecular compounds in BMP were removed by ultrafiltration membrane, and the components were separated by DEAE-52 cellulose column to obtain the refined polysaccharide extract with uniform molecular weight and few impurities. The sample structure was identified indicating that the sugar content was 58.3%, the reducing sugar content was 6.4%, and the protein content was 18.0%. The analysis of monosaccharide fractions showed that the sample mainly consisted of 53.8% glucose, 17.9% galactose, 15.5% mannose, 4.7% rhamnose, 3.5% arabinose, and 2.2% xylose. Through the full-wavelength scanning of UV spectrum, it was found that a small number of proteins in the polysaccharides were covalently bonded. Many characteristic absorption peaks of polysaccharides can be identified by FT-IR spectrum scanning.

Through pathological sections and biochemical indexes, it was verified that intraperitoneal injection of NDMA could successfully establish the model of hepatic fibrosis, which was characterized by weight loss, yellowing hair and fibrous lesions of liver tissue. Low-dose and medium-dose administration of BMP can effectively restore the vitality and body weight of rats, reduce the pathological changes of liver tissue, and reduce the levels of serum enzymes and inflammatory factors. The dosage of BM is very critical, the appropriate dose has a better therapeutic effect than positive drug, but too low dose cannot play a sufficient therapeutic effect, too high dose will increase the burden of rat liver and is not conducive to the recovery of liver fibrosis.

The safe dose range of BM was studied by using BRL-3A cells. LPS activated HSCs-T6, and the cells were observed as fibroblasts under microscope, which marked the success of the model of hepatic fibrosis *in vitro*. The results showed that BMP could inhibit the proliferation of HSCs-T6 induced by LPS and decrease the levels of TGF-β1, IL-1β and TNF-α, indicating that BMP has a good effect on anti-hepatic fibrosis and relieving related inflammation.

BM is often used as a drug for the treatment of liver disease in southwestern China. Our group had previously studied the anti-liver fibrosis activity of flavonoids, triterpenoid saponins, lignans, sesquiterpenoids, phenolic acid and long-chain aliphatic acid compounds in BM, and this article further verified that polysaccharides in this plant also have good anti-liver fibrosis effect, which improved the group’s research about this plant. Combined with the previous work in the laboratory, it can be fully demonstrated that BM can synergistically exert anti-liver fibrosis effects through a variety of substances, including triterpenoid saponins, lignans and polysaccharides, which fully reveals its potential for application in the treatment of liver disease.

## 5 Conclusion

In summary, we extracted polysaccharides from the entire plant of BM. Through the separation and analysis of BMP, it was found that BMP was mainly composed of 8 monosaccharides and a small amount of protein. We constructed *in vivo* and *in vitro* liver fibrosis models by NDMA-induced rats and LPS-activated HSCs-T6, and investigated the mechanism of BMP against liver fibrosis. These works have proved that polysaccharides in BM had potential ability to resist liver fibrosis.

## Data Availability

The original contributions presented in the study are included in the article/Supplementary Material, further inquiries can be directed to the corresponding author.

## Supplementary material

SUPPLEMENTARY FIGURE S1 Effects of different concentrations of LPS on the proliferation rate of HSCs-T6. **p* <0.05, ***p* <0.01, versus control group.
