# Supplementary material for: Isolation and purification of polysaccharides from Bupleurum marginatum Wall.ex DC and their anti-liver fibrosis activities
Source: Front Pharmacol. 2024 Mar 21;15:1342638. doi: 10.3389/fphar.2024.1342638 (PMC10991770; doi:10.3389/fphar.2024.1342638)
Supplement: Supplementary file 1 [file Table1.DOCX]

Supplementary Table 1. The experimental schedule of BMP anti-liver fibrosis induced by NDMN.

| group | treatment | | after-treatment |
| --- | --- | --- | --- |
|  | injection | gavage |  |
| regular group  Control | intraperitoneally injected saline，0.1mL/100g，first 3 days /week | saline, 1mL/100g/day | After  4 weeks, the rats were sacrificed under anesthesia. |
| model group  Model | intraperitoneally injected 1% NDMN，0.1mL/100g，first 3 days /week | saline, 1mL/100g/day |  |
| positive drug group  PC | intraperitoneally injected 1% NDMN，0.1mL/100g，first 3 days /week | 5 mg/mL Silymarin suspension,  1mL/100g/day (0.05g/kg) |  |
| BMP low dose group  L-BMP | intraperitoneally injected 1% NDMN，0.1mL/100g，first 3 days /week | 10 mg/mL BMP,  1mL/100g/day (0.1g/kg) |  |
| BMP medium dose group  M-BMP | intraperitoneally injected 1% NDMN，0.1mL/100g，first 3 days /week | 20 mg/mL BMP,  1mL/100g/day (0.2g/kg) |  |
| BMP high dose group  H-BMP | intraperitoneally injected 1% NDMN，0.1mL/100g，first 3 days /week | 40 mg/mL BMP,  1mL/100g/day (0.4g/kg) |  |
