# Supplementary figures and images for: Isolation and purification of polysaccharides from Bupleurum marginatum Wall.ex DC and their anti-liver fibrosis activities
Source: Front Pharmacol. 2024 Mar 21;15:1342638. doi: 10.3389/fphar.2024.1342638 (PMC10991770; doi:10.3389/fphar.2024.1342638)

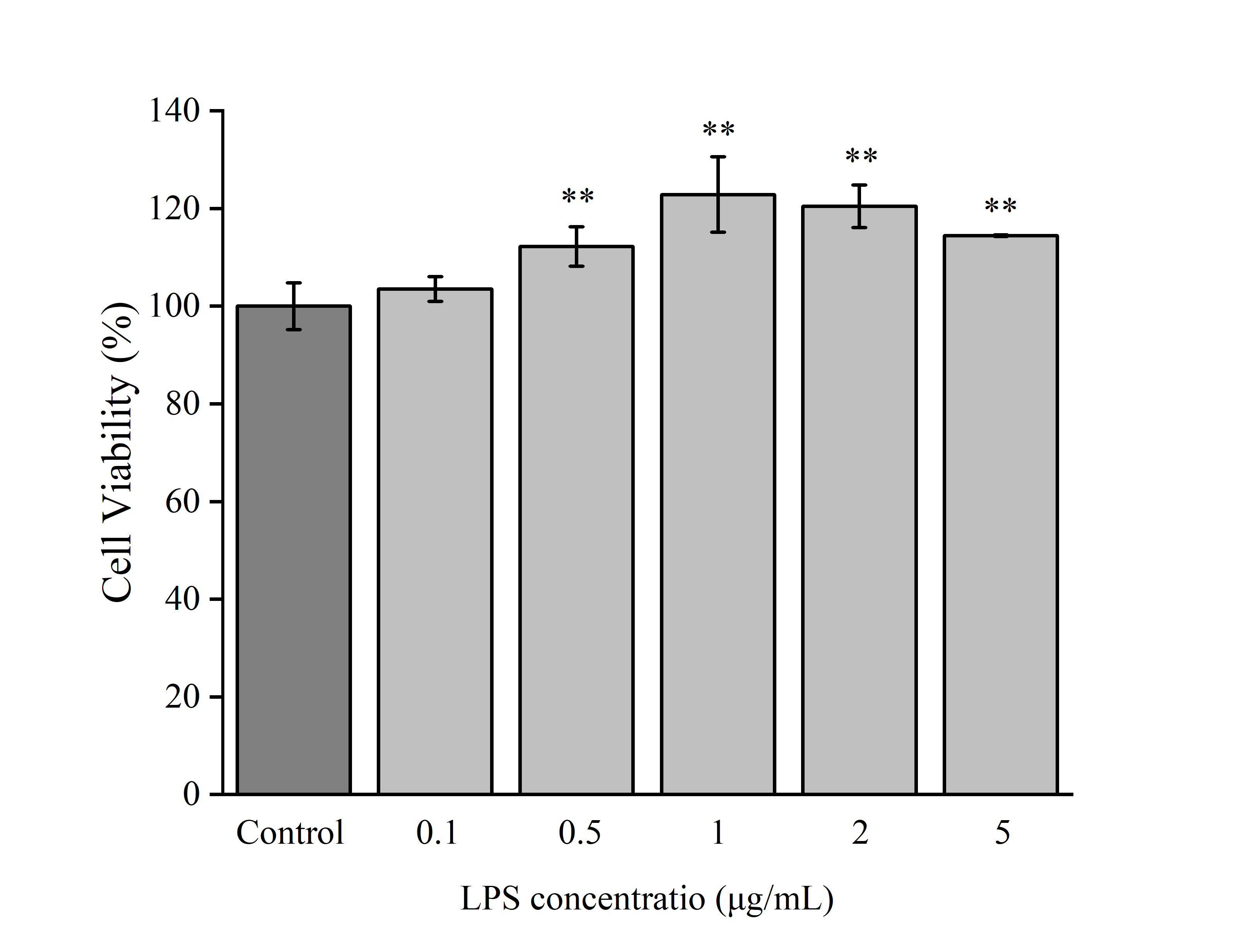

Supplement: Supplementary file 2 [file Image1.TIF]
