# Supplementary material for: Isolation and purification of polysaccharides from Bupleurum marginatum Wall.ex DC and their anti-liver fibrosis activities
Source: Front Pharmacol. 2024 Mar 21;15:1342638. doi: 10.3389/fphar.2024.1342638 (PMC10991770; doi:10.3389/fphar.2024.1342638)
Supplement: Supplementary file 3 [file Table2.DOCX]

Supplementary Table 2. Monosaccharide Component Analysis Standard Sequen.

| Order Number | Name | ppm | Abbreviation | Peak Time (min) | Peak Area |
| --- | --- | --- | --- | --- | --- |
| 1 | Fucose | 5 | Fuc | 5.809 | 16.577 |
| 2 | Galactosamine hydrochloride | 3 | GalN | 10.450 | 22.268 |
| 3 | Rhamnose | 5 | Rha | 10.934 | 8.806 |
| 4 | Arabinose | 3.7 | Ara | 11.442 | 14.329 |
| 5 | Glucosamine hydrochloride | 5 | GluN | 12.884 | 29.828 |
| 6 | Galactose | 5 | Gal | 14.159 | 15.759 |
| 7 | Glucose | 5 | Glu | 15.992 | 19.255 |
| 8 | N-Acetyl-D glucosamine | 5 | GluNAc | 17.609 | 11.589 |
| 9 | Xylose | 5 | Xyl | 18.442 | 18.943 |
| 10 | Mannose | 5 | Man | 19.025 | 16.062 |
| 11 | Fructose | 15 | Fru | 21.334 | 9.535 |
| 12 | Ribose | 10 | Rib | 23.450 | 23.503 |
| 13 | Galacturonic acid | 5 | GalA | 45.050 | 6.059 |
| 14 | Guluronic acid | 10 | GulA | 45.675 | 10.258 |
| 15 | Glucuronic acid | 5 | GluA | 48.059 | 5.180 |
| 16 | Mannuronic acid | 10 | ManA | 50.642 | 8.898 |
